# Plasmonic evolution of atomically size-selected Au clusters by electron energy loss spectrum

**DOI:** 10.1093/nsr/nwaa282

**Published:** 2020-11-25

**Authors:** Siqi Lu, Lin Xie, Kang Lai, Runkun Chen, Lu Cao, Kuojuei Hu, Xuefeng Wang, Jinsen Han, Xiangang Wan, Jianguo Wan, Qing Dai, Fengqi Song, Jiaqing He, Jiayu Dai, Jianing Chen, Zhenlin Wang, Guanghou Wang

**Affiliations:** National Laboratory of Solid State Microstructures, Collaborative Innovation Center of Advanced Microstructures, and School of Physics, Nanjing University, Nanjing 210093, China; Department of Physics, Southern University of Science and Technology, Shenzhen 518055, China; Department of Physics, National University of Defense Technology, Changsha 410073, China; Institute of Physics, Chinese Academy of Sciences and Beijing National Laboratory for Condensed Matter Physics, Beijing 100190, China; School of Physical Sciences, University of Chinese Academy of Sciences, Beijing 100049, China; National Laboratory of Solid State Microstructures, Collaborative Innovation Center of Advanced Microstructures, and School of Physics, Nanjing University, Nanjing 210093, China; National Laboratory of Solid State Microstructures, Collaborative Innovation Center of Advanced Microstructures, and School of Physics, Nanjing University, Nanjing 210093, China; School of Electronic Science and Engineering and Collaborative Innovation Center of Advanced Microstructures, Nanjing University, Nanjing 210093, China; Department of Physics, National University of Defense Technology, Changsha 410073, China; National Laboratory of Solid State Microstructures, Collaborative Innovation Center of Advanced Microstructures, and School of Physics, Nanjing University, Nanjing 210093, China; National Laboratory of Solid State Microstructures, Collaborative Innovation Center of Advanced Microstructures, and School of Physics, Nanjing University, Nanjing 210093, China; Division of Nanophotonics, CAS Center for Excellence in Nanoscience, National Center for Nanoscience and Technology, Beijing 100190, China; National Laboratory of Solid State Microstructures, Collaborative Innovation Center of Advanced Microstructures, and School of Physics, Nanjing University, Nanjing 210093, China; Department of Physics, Southern University of Science and Technology, Shenzhen 518055, China; Department of Physics, National University of Defense Technology, Changsha 410073, China; Institute of Physics, Chinese Academy of Sciences and Beijing National Laboratory for Condensed Matter Physics, Beijing 100190, China; School of Physical Sciences, University of Chinese Academy of Sciences, Beijing 100049, China; Songshan Lake Materials Laboratory, Dongguan 523808, China; National Laboratory of Solid State Microstructures, Collaborative Innovation Center of Advanced Microstructures, and School of Physics, Nanjing University, Nanjing 210093, China; National Laboratory of Solid State Microstructures, Collaborative Innovation Center of Advanced Microstructures, and School of Physics, Nanjing University, Nanjing 210093, China

**Keywords:** atomically precise, gold cluster, plasmonic response, electron energy loss spectroscopy, full-scale evolution

## Abstract

The plasmonic response of gold clusters with atom number (*N*) = 100–70 000 was investigated using scanning transmission electron microscopy-electron energy loss spectroscopy. For decreasing *N*, the bulk plasmon remains unchanged above *N *= 887 but then disappears, while the surface plasmon firstly redshifts from 2.4 to 2.3 eV above *N *= 887 before blueshifting towards 2.6 eV down to *N *= 300, and finally splitting into three fine features. The surface plasmon's excitation ratio is found to follow *N*^0.669^, which is essentially *R*^2^. An atomically precise evolution picture of plasmon physics is thus demonstrated according to three regimes: classical plasmon (*N *= 887–70 000), quantum confinement corrected plasmon (*N *= 300–887) and molecule related plasmon (*N *< 300).

## Teaser

Through the plasmonic investigation of atomically-precision gold nanoparticles, a complete evolution picture linking classical solid-state physics to quantum confinement corrected plasmon and to quantized molecular-like physics is constructed with two atomic precision transition points.

## INTRODUCTION

As an elementary type of collective excitation, plasmon has dominated the optical properties of metals ever since the first experiments were conducted in this area [1], and further interest then arose following the emergence of nanotechnology [2], and in connection with explanations of the Lycurgus Cup. Intense efforts have led to the discovery of some striking behavior, including the existence of hot spots with field enhancement [3], coupling-induced optical shifts [4] and geometrically influenced plasmon absorption [5], as well as potential applications such as biological labeling [6], infrared waveguides [7], cavity [8] and quantum-dot [9] displays. The size-dependence of nanoparticle plasmons is of key interest in studies of this type [10], given that it not only provides reliable nanoparticles with a standard optical response for subsequent assembly and optical operation [11,12], but it is also the only means of reaching a unified understanding in the broad physics that spans from solid state plasmon in large particles [13], to mesoscale and atomic/molecular scale plasmon in particles with countable atoms [14]. By reducing the number of atoms, it is possible to track the evolution of the classical plasmon model to the quantum corrected model. A number of concepts, including quantum plasmon [10,15,16] and electron spill-out effects [17], has emerged during these size-dependent studies. The smallest particles with countable atoms will show quantized molecule-like behaviors [18], where the electrons may even be totally localized and plasmonic excitation seems completely precluded [19]. Controversy also exists on such interesting questions as the division between the nanoparticle and molecules [12,14], and the physics of mesoscopic and microscopic plasmonic evolution [20]. What is more, the inconsistent experimental process, including the temperature, surroundings, geometry and so on brings more controversy. A unified understanding covering the small and large size limits [21], namely macro/meso/micro scales with sufficiently atomic precision, is thus required.

To try to shed some light on these issues, we prepared mass-selected gold clusters Au_100–70 000_ and measured their plasmonic evolution by electron energy loss spectroscopy (EELS) with a scanning transmission electron microscope (STEM) (experimental details are discussed in the Methods section). Two peaks were identified at the center and edge of the clusters, allowing us to study the physics of the evolution of their atom-number dependence. Three regimes with distinct plasmonic physics were observed. Au_887_ was found to be at the boundary between the classical plasmon of nanoparticles and the quantum confinement corrected plasmon (QCC plasmon). The plasmon related to quantized molecular energy levels (molecular plasmon) arises below Au_300_ and was found to be superimposed on coherent single-electron transitions.

## ACQUISITION OF HIGH-QUALITY PLASMONIC SIGNALS OF INDIVIDUAL CLUSTERS BY STEM-EELS

**Figure 1.**
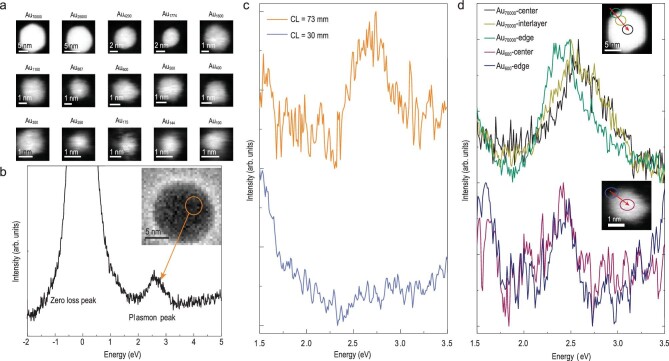
Acquisition of high-quality plasmonic signals of individual clusters using STEM-EELS. (a) EELS mapping of atomically precise gold clusters from 70 000 to 100 atomic numbers. (b) The EEL spectra acquired from the orange spots of an individual gold nanoparticle as shown in the inset. Plasmon feature of gold at ∼2–3 eV can be probed. (c) EEL spectra of Au_1100_ cluster at different CL. When CL = 73 mm (the orange curve), we observe a plasmon resonance peak while there is no obvious signal when CL = 30 mm (the blue curve). (d) The EEL spectra of individual gold atomically precise clusters collected from different positions as marked by spots with the same colors in the inset. For Au_70000_, both BP and SP can be excited in the center, while only SP can be excited at the edge. However, for a small atomic cluster Au_600_, the spectra obtained from the center and edge are similar.

Micrographs of gold clusters with atom numbers *N* from 100 to 70 000 are shown in Fig. 1a. A 700-fold increase in *N* only equals around an order of magnitude change in diameter, especially for small

*N*, where the plasmon peak changes considerably, as described below. This indicates the necessity of this atomically precise *N*-dependent plasmonic study. An incident high-energy electron will excite the plasmonic resonance of a metallic nanoparticle through an inelastic process [22]. A typical electron energy loss spectrum is shown in Fig. 1b, as obtained from a gold nanoparticle in the inset, where a gold plasmonic peak can be seen clearly at 2–3 eV [23]. The beam-focusing configuration was optimized as shown in Fig. 1c for signal optimization. The plasmonic peak increased tens of times when the camera length (CL) was changed from 30 to 73 mm, and this CL is still very small in consideration of dipole approximation to satisfy the existing surface dipole mode for very small nanoparticles [24]. The experimental results provide a satisfactory spatial resolution, as shown in Fig. 1d. For an Au_70 000_ cluster, the green curve (particle edge) is assigned to the peak of the surface plasmon (SP) (∼2.4 eV) [25], while the black curve (particle center) is assigned to the combination of SP and the peak of the bulk plasmon (BP) (∼2.7 eV) [26]. In a smaller particle Au_600_, no obvious difference can be seen between the spectra from the center and edge of the particle, and it seems that only the peak of the SP remains.

## *N*-DEPENDENT EVOLUTION OF THE PLASMON PEAKS

**Figure 2.**
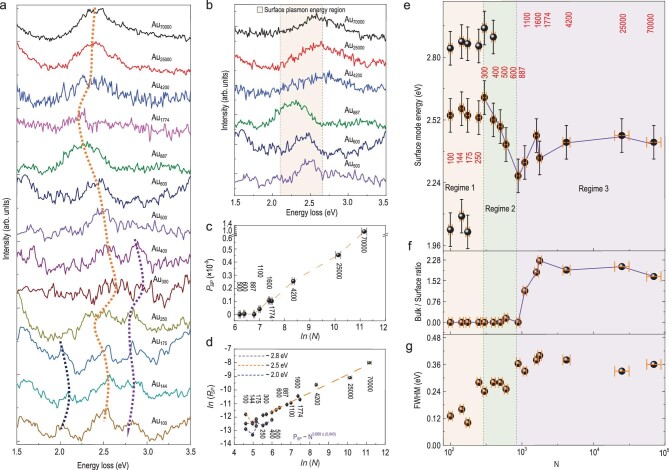
*N*-dependent evolution of the plasmon peaks. (a) The peak of SP evolution of the energy loss spectra collected at the edge of all the gold clusters from Au_70000_ to Au_100_, where the dashed line shows the variation of the center of the peak of SPs. (b) Evolution of central excited plasmons from Au_70000_ to Au_500_. (c) The excitation probability of BP (*P_BP_*) plotted against *ln* (*N*). It drops to nearly zero at *N *= 887. (d) Scaling law of the excitation probability of SP (*P_SP_*) in a *ln* (* P_SP_*)*  ∼  ln(N)* double logarithmic coordination. (e–g) *N*-dependent evolution of the peak of SP position (e), ratio of the excitation probability of BP and SP (*P_BP_/P_SP_*) (f) and the FWHM of the peak of SPs (g). Three characteristic regimes of gold cluster can be classified, including a slight redshift of the peak of SP in regime 3; the diminishing of BP and monotonic blueshift of the peak of SP in regime 2, and multiple peaks in regime 1.

The peak of the SPs collected at the edge of all the gold clusters is shown in Fig. 2a, where the dashed line indicates the variation of the center of the peak. The peak of SP shows a very slight and slow redshift from 2.4 to 2.3 eV with decreasing *N* from 70 000 to 887. With the further decrease of *N*, beyond this point, the peak of the SP shows a gradual blueshift from 2.3 to 2.7 eV before new modes arise for *N* < 300. Three fine features (1.9–2.1 eV, 2.45–2.6 eV, and 2.8–2.95 eV, respectively) arise for the smallest cluster down to *N* ∼ 100. The evolution of the central excited plasmon energy loss spectra is shown in Fig. 2b, in which the peaks of both SP and BP can be detected. The excited plasmon peaks remain steady when *N* decreases from 70 000 to 1100, then the part located at about 2.7 eV disappears abruptly for

smaller clusters with *N* < 887 while the remaining peak exhibits blueshift. The characteristics of the remaining peak appear in the same region, exhibit the same blueshift as the peak of SP, and show no difference between the central and edge excitation (Fig. 1d), which must therefore originate from the peak of SP. We are thus convinced that the peak of BP disappears for all clusters with smaller *N* and therefore show the data from edges for better signal to noise ratios.

It was expected that the spectral yield of the clusters would decrease with the reduction in cluster size, which was indeed observed during the measurements. We calculated the excitation probability of BP (*P_BP_*) by dividing the area of the peak of BP by the area of the zero-loss peak [27]. As shown in Fig. 2c, *P_BP_* decreases by several orders of magnitude with the decrease of *N*, and falls to around zero after *N* ∼ 887, thus confirming the disappearance of the BP mode at Au_887_. Through a similar method we also obtained the excitation probability of the surface plasmon (*P_SP_*) from the boundary area of clusters [27]. From the *ln–ln* framework in Fig. 2d, it is clear that all the data fall scattered around a straight line with a slope of 0.669 ± 0.043, implying a simple power law of the form *P_SP_ ∼ N*^0.669 ±^^ ^^0.043^. Assuming that the volume of an atom in the clusters does not change much, *N* is proportional to *R^3^* (the radius: *R*), i.e. *P_SP_ ∼ R*^2.007 ±^^ ^^0.129^, which means that *P_SP_* depends on the surface area of the gold clusters. This confirms the surface origin of the observed peak of SP. The full width at half maximum (FWHM) of the peak of SP is also given in Fig. 2g, showing a large decrease for small clusters.

Figure 2e–g shows the three characteristic regimes separated by two gray bold vertical dashed lines. In regime 3 (*N ∼* 887–70 000), the positions of the peak of SP exhibit a very slight redshift with decreasing *N*, while the peak of BP remains unchanged and the FWHM remains at a high value of about 0.36 eV. In regime 2 (*N ∼* 300–887), the position of the peak of SP exhibits a steady blueshift with decreasing *N*, while the peak of BP disappears altogether and the FWHM stays at a high value of about 0.26 eV. In regime 1 (*N ∼* 100–300), the peak of SP is replaced by three fine features with a much smaller FWHM, which is close to the FWHM of the EELS zero-loss peak. The physics of the three regimes is discussed below.

## FROM ELECTRON-BOUNDARY SCATTERING MODIFIED CLASSICAL PLASMON TO QCC PLASMON AT Au_887_

**Figure 3.**
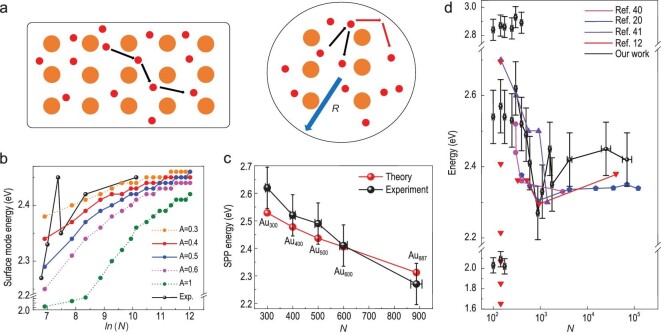
From electron-boundary scattering modified classical plasmon to QCC plasmon below Au_887_. (a) Schematic diagram of electron scattering in an infinite bulk metal (left) and a finite cluster with diameter R (right). The large yellow circles represent the positive ion background while the small red ones represent the ‘free’ electrons. In a finite cluster, the electron-boundary scattering becomes significant. (b) Fitting the large-scale redshift of the peak of SP (experimental data: black, *N *= 887–70 000) with the classical plasmon theory after considering the boundary scattering. Two fitting lines marked in red and blue with A = 0.4 and 0.5, respectively, show good agreement. (c) Fitting the blueshift of the peak of SP (*N *= 300–887) using the formula considering the quantum confinement effect. (d) Comparison of our work with some recent literature. Data are copied from references [12,20,40,41]. The y-axis we label ‘Energy’ here as in [12] ligand-protected Au_144_ cluster is identified to be the small molecule.

According to classical plasmonic physics, the polarizability of the dipole resonance of the localized surface plasmon is determined by the dielectric functions [28]. For large nanoparticles without any obvious difference in electronic structure from those of bulk metals, the reduction in the size only introduces extra boundary scattering for free electrons in the metal, besides the Coulomb scattering between electrons [29], as shown in Fig. 3a. Hence the dielectric function can be formulated [30] by:

(a) $$\begin{eqnarray*}
\varepsilon (\omega ) &=& {\varepsilon _\infty } - \frac{{\omega _p^2}}{{\omega (\omega + i\gamma )}}\nonumber\\ &=& {\varepsilon _\infty } - \frac{{\omega _p^2}}{{\omega (\omega + i{\gamma _{bulk}} + \frac{{iA{v_F}}}{R})}},
\end{eqnarray*}$$

where ϵ_∞_ is incorporated into the dielectric function considering background electron screening at high frequency. In essence, $\gamma = {\gamma _{bulk}} + \frac{{A{v_F}}}{R}$ represents a correction to the electron scattering probability by boundary scattering. *v_F_* is the Fermi velocity of free electrons in gold nanoparticle. *A* is an empirical constant reflecting the details of the scattering processes and it takes a value from 0.1 to 2 [1,31]. As shown in Fig. 3b, the experimental data of the *N*-dependent peak of SP is fitted using the above formula, which reveals good agreement when A = 0.4 or 0.5, between the reported values 0.25 [32] and 0.7 [33], and we confirm the contribution of the boundary scattering as influenced by the size and surface electron density of the cluster [1].

The electronic conduction band, valid at macroscopic scales, breaks down with some gaps when the dimensions are small enough. This quantum confinement effect makes the classical Drude model for the dielectric function invalid [34]. In our experiment the quantum confinement is dominated by smaller *N* (∼887), given that there are so few atoms [35]. The resulting failure of the conduction band of the gold nanoparticles naturally leads to the observed disappearance of the peak of BP [36]. However, the surface electrons are more diffusive, which has a stronger softening effect on the quantization of the surface mode, resulting in a quantum confinement corrected SP with fewer electronic or plasmonic transitions. The permittivity of the Au clusters can be calculated using the Drude model modified with quantum confinement effects [10]. The total permittivity ϵ is the sum of the permittivity of free electron transitions in the quantized conduction band and the frequency-dependent permittivity ϵ_inter_ of interband transitions between the d bands and the higher conduction bands [25]. For the simplest case, we consider only the strongest transitions that emanate from states at the Fermi surface and obtain an approximate solution in a simplified format [37] $\omega _s^2 = \omega _p^2(\frac{{{\omega _{qm}}^{\rm{2}}{\rm{ - }}{\gamma ^2}}}{{\omega _p^2}} + \frac{1}{{\varepsilon _{{\mathop{\rm int}} er}^{{\mathop{\rm Re}\nolimits} }({\omega _s}) + 2{\varepsilon _m}}})$, which is valid for a defined size-dependent dipole transition ω_qm_ at the Fermi surface, representing the quantized conduction band [38], and the scattering frequency γ is given by the same definition as above. In a simple box model [37,38] the corresponding quantum induced blueshifted energy $\hbar{{\rm{\omega }}_{{\rm{qm}}}}\,\,{\rm{ = \,\,}}\hbar {{\rm{\omega }}_{\rm{p}}}\frac{{{{\rm{R}}_{\rm{0}}}}}{{\rm{R}}}$, where R_0_ can be written in terms of the effective free-electron density parameter r_s_ as${R_0}=1.{\rm{1}}{a_0}\sqrt {{r_s}} $, and a_0_ is the Bohr radius. Good agreement can be seen in Fig. 3c. The corresponding energy gap at the Fermi level obtained by Kubo [39] ranges from about 5 meV for Au_887_ to 20 meV for Au_300_, confirming the transition from the classical plasmon to QCC plasmon when N ∼ 887–300. We note that Au_887_ is very small, indicating hardly any quantum effect for most gold nanoparticles as they are larger than Au_887_, and most nanoplasmonic designs in industrial nanofabrication can currently be tackled based on classical electromagnetism and the dielectric function. We gather data from recent literature in Fig. 3d as a comparison with our work. A unified investigation with atomic precision can show many more evolution details, especially in the range of hundreds of atoms where drastic changes happen.

## SUPERIMPOSED TRANSITIONS BETWEEN QUANTIZED MOLECULE-LIKE ELECTRONIC STRUCTURES (*N* < 300)

With only a few dozens to hundreds of atoms, the bulk electronic structure is expected to give way to a complex molecular one because of its quantum nature, and some serious debates have been focused on the nature of the electronic response of these small clusters [42]. As seen in Fig. 2e, for *N* < ∼300, three fine structures arise which lie beyond our traditional understanding of plasmon physics with a single spectroscopic feature [43]. The FWHM of the new features are several times narrower, indicating that these might be from some molecular-like electronic structures. To understand the plasmonic physics of these clusters, a real-time time-dependent density functional theory (rt-TDDFT) calculation is performed. The details of the calculations can be found in the Methods section.

**Figure 4.**
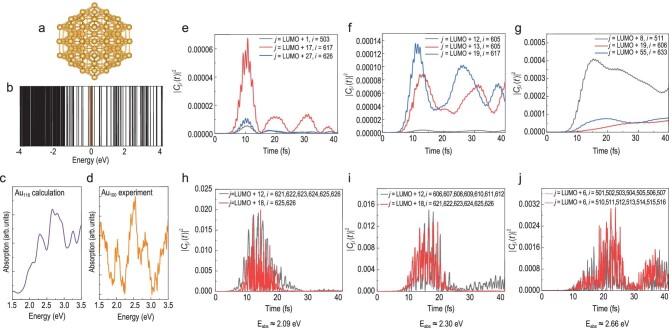
Superimposed transitions between quantized molecule-like electronic structures. (a) Au_116_ Dh geometric structures optimized by DFT. (b) Calculated energy level diagram of Au_116_ Dh structure. The orange solid line at 0 represents the Fermi level. (c and d) Absorption spectra of Au_116_ by rt-TDDFT calculation and experimental EELS spectrum of Au_100_. Theoretical and experimental results both show three split features rather than a single SP peak. (e–j) Transitions ${| {{C_{j,i}}( t )} |^2}$ in the Au_116_ cluster under two kinds of laser field for three different absorption peaks. *j* and *i* denote the unoccupied and occupied energy levels, respectively. The energy difference between *j* and *i* is in the range from E_abs_−0.15 eV to E_abs _+ 0.15 eV. Several important transitions are plotted. The transitions under a weak laser field (e–g) are all single-electron excitation, while plasmon-like behavior (h–j) can be seen for the strong laser field.

We use ion-decahedron (Dh, see Supplementary Fig. S1) Au_116_ as a typical example, the atomic structure of which is optimized as shown in Fig. 4a with calculated electronic structure shown in Fig. 4b. We note that although it exhibits band-like structure around −3 eV, there are obviously discrete energy levels in the (−2, 4 eV) region with the Fermi level set to 0. This clearly shows the molecule-like character of the small gold cluster. We employ the method developed by Wang *et al.* [44] based on rt-TDDFT to analyze the origin of the absorption transition. This method was previously used to investigate the interplay between plasmon and single-electron excitation in Ag_55_ [45].

The optical absorption spectrum was calculated (Fig. 4c), showing a possible spectral contribution in most regions of interest of the three features of small gold clusters (Fig. 4d). We note some difference in the detail between the calculations and experiments. First of all, density functional theory is known to underestimate the energy of excited level, resulting in different positions of spectral peaks compared with the experiments. Secondly, because we used a laser field to excite the electrons, there might have been some difference in the STEM experiments [46].

To analyze the contribution to the spectral peaks, the time-dependent transition coefficients ${| {{C_{j,i}}( t )} |^2}$ for the main absorption peaks were checked (Fig. 4e–j) ($0.0257{\rm{\,\,}}V/{\rm{{\AA}}}$). The transitions can clearly be classified into two different types. One is shown to be rapidly oscillating and another is found to be slowly varying with time evolution, corresponding to plasmon and single-electron transition mode, respectively [45]. When using a laser field weak enough (0.0257 V/Å) to allow the linear excitation of electrons, the experimentally observed features at 2.09, 2.30 and 2.66 eV are single-electron energy level excitations (Fig. 4e–g). However, these change when a higher electrical field is applied. For a laser field of 0.514 V/Å, the transition coefficients of all three experimentally observed features reveal collective electron oscillation modes resulting from coupling between the occupied Kohn-Sham states (Fig. 4h–j). After strong laser action, the collective charge density oscillation can be found at the core of the cluster, which is quite different from the case for a weak laser field (see supplementary Figs S2 and S3). This implies that the occurrence of the plasmon oscillation is found at a stronger laser field. When the laser field is stronger, many-electron excitation can take place, and the excited electron may also exhibit collective oscillations. This reveals that the present

experimental plasmon is some superimposition of single-electron transitions between quantized molecular energy levels. Given that the STEM-EELS is normally used to probe plasmon rather than single-electron transition and in view of the estimated electrical field of ∼10^9^ V/m around 1 nm of a 60 kV electron beam in vacuum in the present study, we are convinced that the three observed features represent molecular plasmon.

## CONCLUSION

In summary, measurements of the *N*-dependent evolution of both the BP and SP of size-selected gold clusters Au_100–70 000_ were obtained using the STEM-EELS approach. Three regimes were observed, each with distinct physics. In the third regime (*N* ∼ 887–70 000), the peak of SP exhibits a slight redshift because of gradually apparent electronic scattering by the particle surfaces. In the second regime (*N* ∼ 300–887), the peak of SP exhibits a steady blueshift and the peak of BP disappears altogether as a result of the quantum confinement effect. In the first regime (*N* ∼ 100–300), the peak of SP is split into three fine features with very small FWHM, indicating the dominance of the molecular energy levels. Thus, a unified set of observations from solid-state classical plasmon physics, QCC plasmon physics and molecular plasmon is demonstrated. This paves the way for new developments in physics and for future applications of nanoplasmonics.

## METHODS

### Sample preparation

Gold nanoclusters were produced using a magnetron sputtering gas phase condensation cluster beam source. A time-of-flight mass filter was used to select clusters of specific atom numbers, offering a mass resolution of M/ΔM ≈ 50. The mass-selected gold clusters were focused into the deposition chamber under high vacuum conditions (10^−5^ – 10^−4 ^Pa), and were deposited onto the ultra-thin carbon film (∼3 nm) on TEM grids at a soft-landing energy of <0.5 eV/atom.

### STEM-EELS collection and data processing

Spectroscopic analysis of the deposited gold clusters was performed with a FEI Titan transmission electron microscope at 60 kV in STEM mode, with an imaging spatial resolution of ∼0.30 nm, an energy dispersion of 0.01 eV per pixel and an EELS zero-loss peak (ZLP) full-width at half maximum of 0.12–0.13 eV. An electron energy of 60 kV was found to have higher excitation probability compared with the use of 300 kV electron energy. Each cluster was mapped in a fully covered square box evenly divided into 40 × 40 square pixels and the dwell time of electron beam on each lattice was 0.001 s to minimize beam damage and drifting of clusters. All the clusters in our experiment shared the same measured parameters in STEM-EELS apart from amplification factor.

### Details of the first principles calculations

Density functional theory (DFT) and real-time time-dependent DFT (rt-TDDFT) were performed within general gradient approximations (GGA) in Perdew-Burke-Ernzerhof (PBE) implementation [47] using the PWmat code [44,48,49]. The norm-conserving pseudopotential produced by the code ONCVPSP [50] was used and 5d^10^6s^1^ was considered to represent the valence electrons of the Au nanoclusters. The k-space was only sampled with the ${\rm{\Gamma }}$ point. A plane-wave basis set with a cutoff of 45 Ry and vacuum space of at least 10 Å were used in all calculations. The atomic coordinates of the Au nanocluster were optimized until the maximum force of all the atoms was <0.01 eV/Å.

For rt-TDDFT, the N-electron system's time-dependent density is given by $n( {r,t} ) = \mathop \sum \nolimits_{j = 1}^N {| {{\psi _j}( {r,t} )} |^2}$, where ${\psi _j}( {r,t} )$ is the single-electron occupied state.

To solve the time-dependent Kohn-Sham (KS) single-electron equation ${H_{KS}}( {n( {r,t} ),t}) {\psi _j}( {r,t} ) = {\rm{i}}\frac{{\partial {\psi _j}( {r,t} )}}{{\partial t}}$, the KS orbitals ${\psi _j}( {r,t} )$ are expanded by the adiabatic KS orbitals ${\varphi _i}( {r,t} )$: ${\psi _j}( {r,t} ) = \mathop \sum \nolimits_i {C_{j,i}}( t ){\varphi _i}( {r,t} )$. ${C_{j,i}}( t )$ is the expansion coefficient and ${\varphi _i}( {r,t} )$ satisfies ${H_{KS}}( {n( {r,t}), t}) {\varphi _i}( {r,t} ) = {\varepsilon _i}( t ){\varphi _i}( {r,t} )$, where ${\varepsilon _i}( t )$ is the energy of ${\varphi _i}( {r,t} )$.

In our rt-TDDFT calculations, the ionic positions are fixed and $n( {r,t} )$ evolves with a time step of 0.01 fs. A Dirac delta electric pulse polarized in the x direction is applied to obtain the absorption spectrum, in which the total simulation time length is 30 fs. To distinguish the excitation modes, we applied two kinds of laser electric field shaped by a Gaussian wavepacket:

(2.9b) $$\begin{eqnarray*}
{\rm{E(t)}}= {{\rm{E}}_{{\rm{max}}}}{\rm{\,\,sin(\omega t)exp\Bigg(}}\frac{{{\rm{ - (t - }}{{\rm{t}}_{\rm{0}}}{{\rm{)}}^{\rm{2}}}}}{{{{\rm{\sigma }}^{\rm{2}}}}}\Bigg).
\end{eqnarray*}$$

The first reaches maximum intensity E_max_ = 0.0257 V/Å at time t_0 _= 9 fs, and the pulse duration σ is 3.3 fs. For the second, the E_max_ and σ are enhanced to 0.514 V/Å and 6 fs, respectively. $\omega $ is set to the resonant frequency.

## DATA AVAILABILITY

The data that support the findings of this study are available from the corresponding author upon reasonable request.

## Supplementary Material

nwaa282_Supplemental_File
