# Supplementary material for: Plasmonic evolution of atomically size-selected Au clusters by electron energy loss spectrum
Source: Natl Sci Rev. 2020 Nov 25;8(12):nwaa282. doi: 10.1093/nsr/nwaa282 (PMC8972990; doi:10.1093/nsr/nwaa282)
Supplement: nwaa282_Supplemental_File [file nwaa282_supplemental_file.docx]

**SUPPLEMENTARY INFORMATION**

In other recent experimental studies, Au_923_ shows a structure component that ino-decahedron (Dh)＞bulk-favorable cuboctahedron structure (Fcc)＞icosahedron (Ih) [1, 2]. In this work we also observed 3 major structures of Au_600_, namely ino-decahedron, icosahedron and cuboctahedron Au_600_, as shown in Fig. S1a. As for large clusters like the 15 nm ones, the EELS signal is so high that only several individual clusters are enough to acquire the plasmonic signal. We find that these mapping ones are the bulk-favorable cuboctahedron structure.

To be consistent with the EELS mapping condition, the so-called beam shower process is applied to the clusters in STEM for 30 minutes before the image collection. After that we find that most of the Au_600_ clusters are changed to *ino-decahedron structure*, as seen in Fig. S1b. Recent research by R.E. Palmer also claim the transitions to ino-decahedron structures after electron beam irradiation[3]. We should emphasize that the structure statistics was taken in 300 kV STEM condition, which is different from the STEM-EELS condition with 60 kV electron beam. Since with 60 kV electron beam the plasmon excitation probability is high at the cost of the poor structural resolution.

Theoretically small gold clusters with hundreds of atoms are calculated to prefer penta-rotating symmetrical decahedral motif[4]. Also, in our theoretical calculations we found that the *ino-decahedron structure* of small clusters satisfied the experimental diameter data well, as seen in Fig. S1c.


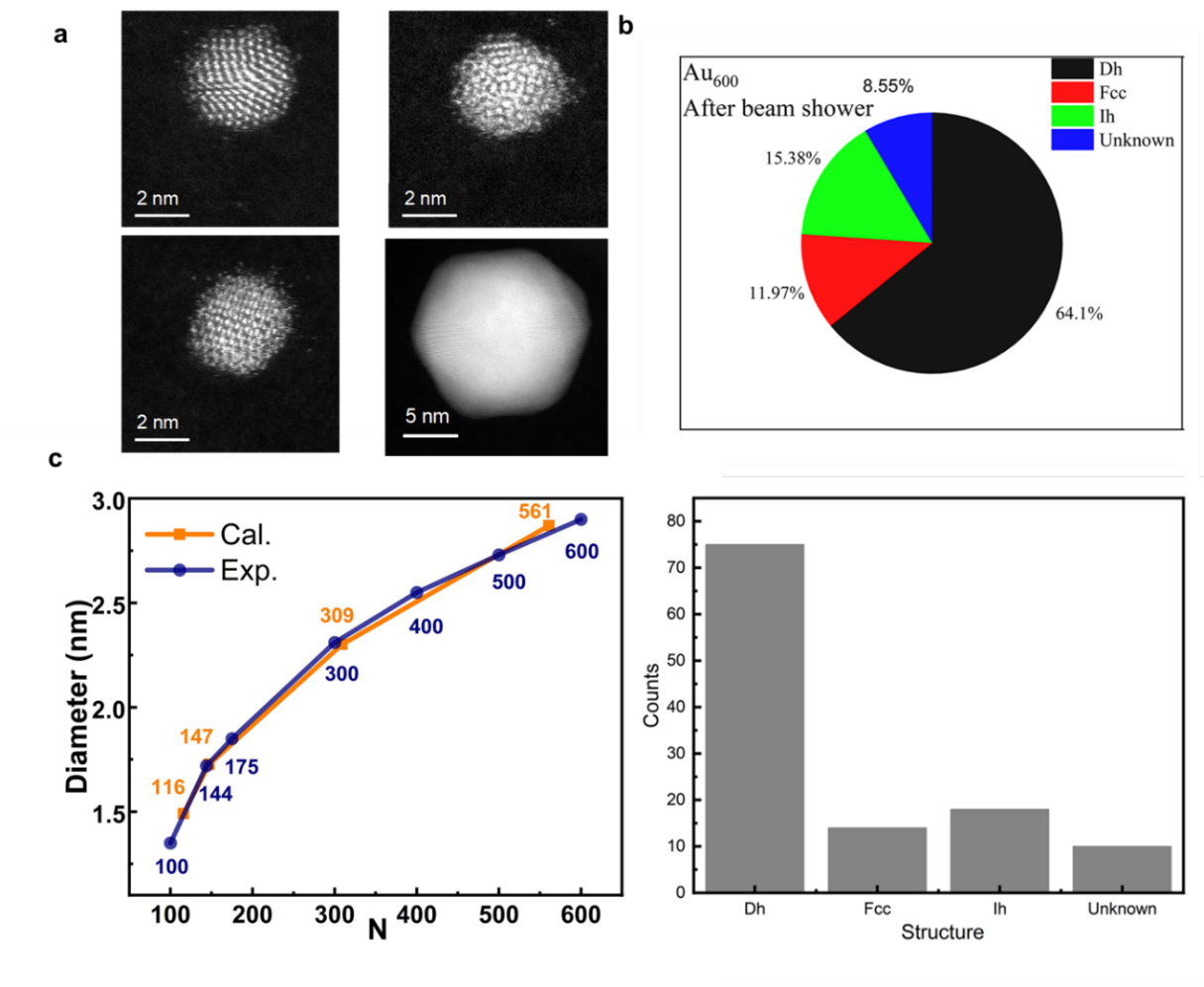


**Supplementary Figure S1**. a. Different structures of gold clusters. ino-decahedron Au_600_ (top left); icosahedron Au_600_ (top right); cuboctahedron Au_600_ (down left) and cuboctahedron 15 nm gold nanoparticles (down right) respectively. b. Structural statistics of Au_600_ clusters after 30 minutes beam shower in pie chart and histogram. c. Comparison of calculated (orange) and experimental (blue) results of ino-decahedron gold clusters.

**Supplementary Figure S2**. Charge density oscillation of Au_116_ Dh under weak laser field. a) The laser field applied in Au_116_ Dh (black line, left part) and the corresponding induced dipole moment (red line, right part). b-e) The top view of charge density difference $\Delta\rho\left( r,t \right)$ which equals $\rho\left( r,t \right)-\rho_{0}\left( r \right)$, where $\rho\left( r,t \right)$ and $\rho_{0}\left( r \right)$ are charge density at time t and the ground state, respectively. The isovalue is 0.0002 e/Å^3^. The corresponding dipole moment is labelled in (a). After the laser fading off, the collective oscillation of electrons is not found.

**Supplementary Figure S3**. Charge density oscillation of Au_116_ Dh under strong laser field. a) The laser field applied in Au_116_ Dh (blue dashed line, left part) and the corresponding induced dipole moment (red line, right part). b-e) The top view of charge density difference $\Delta\rho\left( r,t \right)$. The isovalue is 0.005 e/Å^3^. The corresponding dipole moment is labelled in a). After the laser fading off, the collective oscillation of electrons is observed at the core of the cluster, which means the strong laser field might induce plasmon in the Au_116_ Dh.

**References**

1. Wells DM, Rossi G, Ferrando R*, et al.* Metastability of the atomic structures of size-selected gold nanoparticles. *Nanoscale*. 2015; **7**(15): 6498-503.

2. Plant SR, Cao L, Palmer RE. Atomic Structure Control of Size-Selected Gold Nanoclusters during Formation. *J Am Chem Soc*. 2014; **136**(21): 7559-62.

3. Wang ZW, Palmer RE. Determination of the Ground-State Atomic Structures of Size-Selected Au Nanoclusters by Electron-Beam-Induced Transformation. *Phys Rev Lett*. 2012; **108**(24): 245502.

4. Cleveland CL, Landman U, Schaaff TG*, et al.* Structural Evolution of Smaller Gold Nanocrystals: The Truncated Decahedral Motif. *Phys Rev Lett*. 1997; **79**(10): 1873-6.
